# Predictors of Pressure Injury Development and Clinical Course in ICU Patients: A Retrospective Cohort Study

**DOI:** 10.3390/healthcare14091150

**Published:** 2026-04-25

**Authors:** Elif Kerimoğlu

**Affiliations:** Ministry of Health Ordu State Hospital, 52200 Ordu, Türkiye; ekerimoglu1@gmail.com

**Keywords:** NRS 2002, Braden scale, nutritional screening, pressure injury, risk factors

## Abstract

**Objective**: This study evaluated the relationships between the development and clinical course of pressure injuries (PIs) and neurological status, nutritional risk, and laboratory parameters among patients admitted to a tertiary intensive care unit. **Materials and Methods**: The single-center, retrospective, observational study included 220 patients hospitalized in the intensive care unit for at least 5 days. On the day of admission, Glasgow Coma Scale (GCS), Acute Physiology and Chronic Health Evaluation II (APACHE II), Braden, and Nutritional Risk Screening 2002 (NRS-2002) scores were assessed. Demographic characteristics, comorbidities, need for sedation and vasopressors, and laboratory parameters during the first 24 h (albumin, C-reactive protein, lactate, D-dimer) were analyzed. Factors independently associated with new PI development and clinical improvement were identified using binary logistic regression. **Results**: New PIs developed in 25% of patients. Patients with PI progression were older and had lower GCS and Braden scores, higher NRS-2002 scores, lower albumin levels, and higher D-dimer levels (*p* < 0.05). In multivariable analysis, low GCS (OR = 0.824), presence of comorbidity (OR = 2.327), and a high NRS-2002 risk level were independent predictors of new PI development. The model’s discriminative ability was acceptable (AUC = 0.756). Among patients with existing PIs, NRS-2002 score (OR = 0.450) and age (OR = 1.058) were independently associated with clinical improvement in an exploratory multivariable model. **Conclusions**: NRS-2002 was the only variable independently associated with both new PI development and the clinical improvement of existing lesions, underscoring the central role of nutritional risk assessment in ICU-based PI prevention and prognosis.

## 1. Introduction

Pressure injury (PI) is defined as a localized injury to the skin and underlying tissue that results from a combination of pressure and shear forces, frequently caused by contact with support surfaces or medical devices [1].

The risk of PI development in patients requiring intensive care is further increased by neurological disorders, high or low body mass index (BMI), nutritional deficiencies, immobilization, hemodynamic instability, mechanical ventilation, use of vasopressor agents, and device-related pressure [2,3,4]. PIs may lead to infection and sepsis, increase the need for corrective surgery, prolong hospital length of stay, and increase mortality [5]. PIs are observed at approximately four-times higher rates in ICU patients compared with non-ICU patients [6]. The global prevalence of PIs in the ICU setting has been reported as 26.6%, and 16.2% of these were reported to have developed de novo during follow-up [7].

The guideline from the European Pressure Ulcer Advisory Panel, National Pressure Injury Advisory Panel, and Pan Pacific Pressure Injury Alliance (EPUAP, NPIAP, and PPPIA, 2019) recommends the use of structured risk-assessment tools, supported by clinical judgment, in the assessment of PI risk [5]. The purpose of risk-assessment tools is to identify patients who are vulnerable to PIs and to characterize relevant risk factors [8]. None of the existing tools is considered a gold-standard [9]. The core components of PI prevention are risk assessment and care bundles, and the Braden risk-assessment scale is one of the validated risk-assessment instruments [8]. The Jackson/Cubbin Scale has been proposed as an ICU-specific alternative with potentially superior predictive validity in critical care settings. However, the Braden Scale was selected for this study because it was the instrument routinely used and prospectively recorded in the study unit throughout the observation period, and its application was protocol-mandated for all admissions. Retrospective use of a non-implemented scale would have required score reconstruction from chart data, introducing risk of measurement error. Furthermore, the Braden Scale remains the most widely validated and internationally benchmarked tool in both general and ICU populations, facilitating comparability with existing literature [5,8].

Malnutrition adversely affects immune function, wound healing, overall recovery, and prognosis; nutritional screening is, therefore, considered a fundamental step for appropriate nutritional interventions in critically ill patients [10,11]. Nutritional Risk Screening 2002 (NRS-2002) is a tool that evaluates weight changes, BMI, and disease severity together [10]. The European Society for Clinical Nutrition and Metabolism (ESPEN) recommends the use of the NRS-2002 score to identify nutritional risk in critically ill patients [10]. In the intensive care population in particular, the combined consideration of neurological status (Glasgow Coma Scale, GCS), disease severity (APACHE II), nutritional risk (NRS-2002), and pressure-risk assessment tools has been examined in a limited number of studies with respect to predicting both new PI development and the clinical course of existing PIs. A holistic evaluation of these parameters may contribute to more accurate prediction of PI occurrence and prognosis.

In this study, GCS, APACHE II, Braden risk scale, NRS-2002, and admission-day laboratory data calculated on the day of intensive care admission were examined together with PI stages recorded during hospitalization and follow-up, to investigate the roles of these risk-assessment tools in identifying patients at risk for PI development.

## 2. Materials and Methods

This study was designed as a single-center, retrospective, observational study. Data from 220 patients aged >18 years who were admitted to the Ministry of Health Ordu State Hospital ICU between 1 August 2024 and 1 November 2025 and who had an ICU stay of at least 5 days were included. This threshold was applied to ensure an adequate observation window for PI development and clinical course assessment. To evaluate potential selection bias introduced by this criterion, a sensitivity analysis was performed comparing patients with ICU stays of less than five days to those meeting the inclusion criterion. The ICU was a tertiary-level, mixed medical–surgical intensive care unit, accepting patients with acute medical illness, post-operative care needs, and medical emergencies, but not functioning as a dedicated trauma or neurosurgical unit. Approval for the study was obtained from the Ordu University Non-Interventional Scientific Research Ethics Committee (date: 26 December 2025, decision no: 2025/448). Because retrospective data were used, the requirement for informed consent was waived by the ethics committee.

### 2.1. Study Design

All data were collected retrospectively from the hospital information management system (HIMS), laboratory information system, and nursing care notes. Age, sex, and comorbidities (malignancy; cardiovascular/metabolic; respiratory) were recorded. Comorbidity burden was additionally quantified using the Charlson Comorbidity Index (CCI), which assigns weighted scores to 19 predefined conditions and has been validated for use in critically ill patients. Neurological status on the day of admission was determined using GCS. Disease severity was assessed by the APACHE II score calculated during the first 24 h of ICU admission. Laboratory values measured within the first 24 h (C-reactive protein [CRP], lactate, serum albumin, and D-dimer). Length of stay in hospital and in the ICU, and the use of vasopressors and sedatives were also recorded.

Patients were classified into four groups according to the clinical course of PIs: (1) no PI and none developed; (2) no PI on admission and a new PI developed; (3) existing PI worsened or remained unchanged; and (4) existing PI improved or regressed. Clinical grouping was based on the PI stages and follow-up records documented in the patient files. Deep tissue pressure injury (DTPI), as defined by the NPIAP classification, was not included as a separate category in this study because the retrospective clinical records did not consistently differentiate DTPI from Stage 1 or Stage 2 injuries at the time of documentation. Staging was therefore limited to the four-stage classification to ensure consistency across the data.

PIs were classified according to the 2014 NPUAP/EPUAP clinical practice guideline [12] which defines a four-stage classification system. This staging system is equivalent across the NPUAP (now NPIAP) and EPUAP frameworks, as both organizations jointly developed and endorsed the 2014 guideline. Stage 1 was defined as intact skin with a localized area of non-blanchable erythema. Stage 2 was defined as partial-thickness loss of the dermis. Stage 3 was defined as loss involving the skin and subcutaneous tissue layers. Stage 4 was defined as full-thickness tissue loss. The clinical course of ulcers managed by the wound care team was followed using the decubitus ulcer staging recorded in patient files (Stages I–IV) and nursing electronic records, including photographic follow-up notes.

PI risk was assessed with the Braden Risk Assessment Scale on the day of admission. The Braden scale evaluates six domains: sensory perception, moisture, activity, mobility, nutrition, and friction/shear. Each domain is scored using values that range from 1 to 3 or 1 to 4, depending on the subscale. The maximum possible total score is 23. Risk categories were defined as follows: high risk, <12; moderate risk, 13–15; and mild risk, 16–17 or higher [13,14].

Nutritional risk screening was performed via the NRS-2002 on the day of admission. NRS-2002 evaluates BMI, dietary intake during the previous week, weight loss over the previous three months, and presence of severe disease. Disease severity and deterioration of nutritional status are scored from 0 to 3 (none, mild, moderate, or severe). An additional point is added for patients aged ≥70 years. Total scores range from 0 to 7. Scores of 0–3 indicate low nutritional risk, score of 4 indicates “at risk,” and scores of 5–7 indicate high nutritional risk [15,16].

### 2.2. Statistical Analysis

Statistical analyses were performed using SPSS version 27.0. Continuous variables were presented as median and interquartile range (IQR). Categorical variables were presented as number and percentage [n (%)].

The distribution of continuous variables within groups was assessed with the Shapiro–Wilk test. Because the data did not meet the assumption of normality, nonparametric methods were used for group comparisons. For comparisons involving more than two groups, the Kruskal–Wallis test was applied. For variables that reached statistical significance, Dunn post hoc tests with Bonferroni correction were performed.

Categorical variables were analyzed using the chi-square test. When expected cell frequencies were low, Monte Carlo simulation was used.

Separate binary logistic regression analyses were performed to identify factors independently associated with (a) new PI development and (b) the clinical course of existing PIs. Results were reported as odds ratios (ORs) with 95% confidence intervals (CIs). Model calibration was evaluated with the Hosmer–Lemeshow goodness-of-fit test. Model explanatory power was assessed using Nagelkerke R^2^. Discriminative ability of the models was analyzed by receiver operating characteristic (ROC) curve analysis and area under the curve (AUC) calculation. Multicollinearity in multivariable models was assessed using the variance inflation factor (VIF). In addition, collinearity diagnostics were assessed using condition index values derived from the linear regression framework. The maximum condition index in the models was 25.501, which is below the conventional threshold of 30, indicating no serious multicollinearity. Variance proportions did not concentrate on any single dimension. The strong correlation observed between GCS and the Braden total score (r = 0.828; *p* < 0.001) was addressed by not including both simultaneously in the same model. A two-tailed *p*-value < 0.05 was considered statistically significant.

## 3. Results

A total of 455 ICU patients were screened for inclusion, of which 220 patients with complete data were included and 235 patients with an ICU stay of less than 5 days were excluded. Two patients were transferred from a palliative care unit to the ICU due to acute clinical deterioration requiring organ support; these patients met the inclusion criteria and were therefore retained in the analysis. The demographic and clinical characteristics of the patients included in the analysis are shown in Table 1.

Patients were divided into four groups according to PI status: no PI/none developed (n = 84), new PI developed (n = 55), existing PI worsened/unchanged (n = 61), and existing PI improved/regressed (n = 20). Between-group comparisons were performed using nonparametric tests for continuous variables and chi-square tests for categorical variables.

As shown in Table 1, the median age of the cohort was 74 years (IQR 64–85), indicating that the sample was concentrated in an older age group. Median length of hospitalization was 19 days (IQR 12–34), and median ICU length of stay was 10 days (IQR 6–18.75). Examination of intensive care scores revealed a median APACHE II score of 24 (IQR 20–27), median GCS of 9 (IQR 4–12), median Braden score of 10 (IQR 6–13), and median NRS-2002 score of 4 (IQR 3–5). Median CCI was 7 (IQR 6–9).

Median albumin was 30.7 g/L (IQR 27.35–34.8), median CRP was 73 mg/L (IQR 36–123), median lactate was 1.7 mmol/L (IQR 1.3–2.6), and median D-dimer was 1.58 mg/L (IQR 0.76–3.55).

Almost half of the patients (55.5%) were male, and 53.6% were admitted from the emergency department. The most common reason for admission was respiratory failure (54.1%). Among comorbid conditions, cardiovascular/metabolic diseases were most frequent (50.9%), and the prevalence of malignancy was 13.6%. During the ICU course, 59.5% of patients required sedation and 65.0% required vasopressor support.

The proportion of patients without PIs at admission was 63.2%; this proportion decreased to 45.9% at discharge. An increase was observed particularly in the proportion of advanced-stage (Stage 3–4) PIs. The overall mortality rate was 38.6%.

When PI distribution was examined, 38.2% of patients did not develop a PI, 25.0% developed a new PI, 27.7% had an existing PI that worsened or remained unchanged, and 9.1% experienced improvement.

### 3.1. Comparison of Continuous Variables According to PI Groups

The comparison of continuous variables across PI groups are shown in Table 2.

### 3.2. Post Hoc Comparisons

Dunn–Bonferroni post hoc comparisons for variables that reached statistical significance are presented in Table 3.

When PI groups were compared, age differed significantly across groups (*p* = 0.003). Post hoc analysis showed a significant difference only between the “No PI/none developed” and “Existing PI improved/regressed” groups.

GCS differed significantly among groups (*p* < 0.001). GCS was higher in the “No PI/none developed” group but was lower in the groups with new PI development and with existing PI worsening.

NRS-2002 score was significantly different between the groups (*p* < 0.001). The highest NRS-2002 scores were observed in the “Existing PI worsened/unchanged” group. Among the variables, NRS-2002 most clearly reflected between-group differences.

Braden score also differed significantly between the groups (*p* < 0.001). Braden scores were higher in the “No PI/none developed” group and lower in the “New PI developed” and “Existing PI worsened/unchanged” groups.

Albumin levels differed between groups (*p* = 0.002). Albumin was lower in the “Existing PI worsened/unchanged” group. D-dimer levels also showed a significant difference between the groups (*p* = 0.006). The highest D-dimer values were observed in the “Existing PI worsened/unchanged” group.

Length of hospitalization differed significantly between the groups (*p* = 0.002). Hospitalization was longest in the “Existing PI worsened/unchanged” group.

There were no significant between-group differences in APACHE II, CRP, lactate, ICU length of stay, or the Charlson Comorbidity Index (*p* > 0.05).

Overall, poorer clinical course of pressure injury (i.e., groups with new PI development and with existing PI worsening) was associated with lower GCS, higher NRS-2002 scores, lower Braden scores, lower albumin levels, and longer hospital length of stay. Among these variables, NRS-2002 stood out as the parameter that most distinctly reflected differences between groups.

### 3.3. Correlations Between Clinical Scores

The correlation between GCS and the Braden total score was assessed using Spearman’s rank correlation. A strong positive correlation was observed (r = 0.828; *p* < 0.001), consistent with the known conceptual overlap between neurological status and the sensory perception domain of the Braden scale. Because individual Braden subscale scores were not available as separate variables in the hospital information system, the correlation analysis was performed using the Braden total score.

The correlation between NRS-2002 and the Braden total score was statistically significant but weak (r = −0.190; *p* = 0.005). Because Braden subscale scores were not separately extractable from the medical records, this analysis reflects the relationship between NRS-2002 and the overall Braden composite score rather than the nutritional subscale specifically.

### 3.4. Comparison of Categorical Variables According to PI Groups

The distribution of categorical variables across groups is shown in Table 4.

Comparison of categorical variables according to PI groups is presented in Table 4. No significant association was found between sex and PI group (*p* = 0.742). A near-significant association was observed between the presence of comorbidity and PI groups (*p* = 0.050).

A significant association was found between the source unit on admission and PI groups (*p* = 0.025). Patients admitted from Level 2 ICU had higher rates of PI worsening.

Need for sedation (*p* = 0.014) and need for vasopressors (*p* = 0.037) were both significantly associated with PI groups. The rates of both sedation and vasopressor use were higher in the group that developed new PIs.

Braden risk level differed significantly across PI groups (*p* = 0.015). The high-risk Braden category had a markedly higher proportion of patients with PI worsening.

No significant association was observed between APACHE II risk level and PI groups (*p* = 0.719).

The strongest association was observed for NRS-2002 risk level (*p* < 0.001). The high-risk NRS-2002 category had a substantially higher proportion of patients whose existing PIs worsened.

### 3.5. Factors Associated with New PI Development (Model A)

A multivariable binary logistic regression analysis was performed on the 139 patients who had no PIs at admission to identify independent predictors of new PI development during follow-up. The dependent variable was new PI development (0 = No, 1 = Yes). Independent variables included in the model were GCS, NRS-2002 risk level (reference category: high risk), and presence of comorbidity (reference category: none). Variable selection was based on clinical relevance and the ratio of events per variable (Table 5).

Model calibration was acceptable (Hosmer–Lemeshow *p* = 0.285). The model explained approximately 25% of the variance in new PI development (Nagelkerke R^2^ = 0.254). ROC analysis indicated acceptable discrimination (AUC = 0.756).

GCS was independently associated with new PI development (OR = 0.824; 95% CI: 0.746–0.911; *p* < 0.001). Each one-point increase in GCS reduced the odds of new PI development. The presence of comorbidity increased the risk of new PI development by approximately 2.3-fold (OR = 2.327; 95% CI: 1.016–5.330; *p* = 0.046).

NRS-2002 risk level was an independent predictor (overall *p* = 0.028). Compared with the high-risk NRS-2002 category, the low-risk category had a significantly lower odds of new PI development (OR = 0.263; 95% CI: 0.095–0.728; *p* = 0.010). When NRS-2002 was entered into the model as a continuous variable, it was not statistically significant (OR = 1.033; 95% CI: 0.739–1.444; *p* = 0.849), whereas the effects of GCS and comorbidity remained similar.

### 3.6. Factors Associated with Clinical Course (Model B)

A binary logistic regression analysis was performed to identify predictors of clinical improvement among patients who had PIs at admission. The dependent variable was clinical improvement (0 = No, 1 = Yes), and the analysis was restricted to patients with a PI on admission (n = 81).

To evaluate factors independently associated with clinical improvement, a parsimonious multivariable logistic regression model was constructed including NRS-2002 score, albumin, Braden score, and age (Model B—Exploratory Model) (Table 6).

To evaluate whether the association between NRS-2002 and clinical improvement was independent of other clinically relevant variables, a parsimonious multivariable model was constructed including NRS-2002 score, albumin, Braden score, and age. NRS-2002 score (OR = 0.450; 95% CI: 0.274–0.739; *p* = 0.002) and age (OR = 1.058; 95% CI: 1.008–1.111; *p* = 0.022) were independently associated with clinical improvement, while albumin and Braden score did not contribute significantly (both *p* > 0.05). Model calibration was acceptable (Hosmer–Lemeshow *p* > 0.05) and explanatory power was moderate (Nagelkerke R^2^ = 0.281). Given the small number of events (n = 20), this model should be interpreted with caution and considered exploratory.

### 3.7. Relationship Between Mortality and PI Outcomes

Among patients without a PI at admission (n = 139), mortality was not significantly associated with new PI development (39.8% in survivors vs. 39.2% in non-survivors; χ^2^ = 0.004; *p* = 0.948). Among patients with a PI at admission (n = 81), mortality was similarly not associated with clinical improvement (27.7% improvement rate in survivors vs. 20.6% in non-survivors; χ^2^ = 0.531; *p* = 0.466). These findings suggest that PI development and healing trajectory in this cohort were largely independent of in-hospital mortality.

## 4. Discussion

This study evaluated factors associated with the development of PIs and the clinical course of existing PIs among ICU patients.

In a study of 4499 stroke patients by Liu et al., the incidence of PIs was reported as 15.6% [17]; in the present study the incidence was 25%. Despite differences in incidence, both studies identified low GCS as an independent predictor of PI development, supporting the view that neurological status is a consistent determinant of PI risk.

The higher requirement for sedation observed in the group that developed new PIs supports the role of immobilization in PI pathogenesis. In the NONSEDA trial, sedation was not associated with the number of PIs (*p* = 0.08), although differences in PI localization were reported [18]. This finding suggests that sedation may modulate PI risk by influencing immobilization time and pressure distribution. Although sedation and vasopressor use were significantly associated with PI group distribution in univariate analyses, neither variable was independently associated with new PI development or clinical improvement after adjustment for NRS-2002 score and age in multivariable analyses (sedation: OR = 1.383, *p* = 0.592; vasopressor: OR = 0.714, *p* = 0.586). This suggests that the effects of these clinical interventions may be mediated through, or confounded by, disease severity and nutritional status rather than representing independent causal pathways.

The greater need for vasopressor support among patients who developed new PIs suggests a role for hemodynamic instability in PI pathogenesis. In their retrospective analysis, Woźniak et al. reported a significant association between the need for hemodynamic support and PI development [19]. These findings suggest that PI development involves systemic perfusion impairment and tissue hypoxia in addition to local mechanical factors.

Lower albumin levels observed in patients with PI progression may reflect diminished nutritional reserves and impaired tissue repair capacity. In the study by Merriman et al., albumin remained an independent predictor, whereas CRP and other inflammatory markers did not demonstrate such effects [20]. In the present study, CRP did not differ significantly between groups, which is concordant with the findings of Merriman et al. However, albumin did not retain independent predictive value in our multivariable model. Higher D-dimer levels in the group with PI progression may be related to microcirculatory dysfunction and coagulation activation. Experimental models indicate that sustained mechanical pressure exacerbates ischemia–reperfusion injury, reduces microvascular density, increases oxidative stress, and triggers an inflammatory response [21].

It is notable that APACHE II scores did not differ significantly between groups. A systematic review by Tang reported higher APACHE II scores among patients who developed PIs, with a pooled effect size that was statistically significant (SMD 0.82; 95% CI 0.58–1.06) [22]. However, that review also reported substantial heterogeneity (I^2^ = 91.7%), indicating that the predictive performance of APACHE II for PI risk may vary by clinical context, which could explain why APACHE II did not emerge as an independent predictor in our study.

NRS-2002 scores differed markedly between groups, with the highest scores observed in patients whose PIs progressed. Serpa et al. reported that patients at nutritional risk developed PIs significantly more often [11]. Hiramatsu et al. found that the presence of PIs was independently associated with failure of nutritional support (HR 1.74; 95% CI 1.02–2.98), and low nutritional indices were identified as factors negatively affecting prognosis [23]. NRS-2002 was the only variable associated with both new PI development and the healing of existing lesions, suggesting that nutritional status functions as a determinant of both PI incidence and recovery capacity. Early nutritional risk stratification should, therefore, be considered a central component of PI prevention and prognosis management in the ICU [24].

In our cohort, the median length of hospitalization in the group with worsened/unchanged PIs was 26 days (IQR 14.5–42), whereas it was 14 days (IQR 10–25.8) in the group without PIs; this difference was statistically significant (*p* = 0.002). In contrast, length of ICU stay did not differ significantly between the groups (*p* = 0.196). Montero-Marco et al. reported that elderly patients at risk for PIs are associated with prolonged hospital stays and that hospital-acquired PIs are more frequent at discharge [25]. Early risk-reduction strategies for ICU patients expected to have prolonged hospital stays may be critical to limit PI progression.

Age differed significantly between groups (*p* = 0.003); however, post hoc analysis showed a significant difference only between the “No PI/none developed” and “Existing PI improved/regressed” groups. Notably, the median age in the group with clinical improvement was higher than in the other groups. Itoh et al. showed that in patients aged ≥75 years, the risk of PIs increased in association with clinical factors such as low albumin (OR 0.51), cardiovascular disease (OR 2.10), infection (OR 1.91), and bed dependency (OR 1.85) [26]. These findings suggest that age may act through interactions with frailty, comorbidity burden, and functional dependency rather than as a direct biological risk factor.

A significant association was found between the source unit on admission and PI groups (*p* = 0.025). Patients transferred from Level 2 ICU had a PI worsening rate of 29.5% compared with 11.9% in the group without PIs. Fulbrook et al. reported a markedly higher prevalence of hospital-acquired PIs in ICU patients compared with non-ICU patients (9.6% vs. 2.1%) [6]. In the present study, the higher progression rate observed among patients transferred from Level 2 ICUs supports the notion that PI risk is dynamic and may change throughout the continuum of care.

### 4.1. Predictive Model for New PI Development (Model A)

In the multivariate model, GCS, NRS-2002 risk level, and presence of comorbidity were independent predictors of new PI development. Each one-point increase in GCS reduced the odds of new PI development (OR = 0.824; 95% CI 0.746–0.911; *p* < 0.001).

NRS-2002 risk level was also an independent predictor (overall *p* = 0.028). Compared with the high-risk category, the low-risk category had a significantly lower likelihood of developing a new PI (OR = 0.263; 95% CI 0.095–0.728; *p* = 0.010). The finding that categorical risk strata (rather than continuous score) were significant suggests that clinical threshold values may be more informative than a strictly linear relationship. This result is consistent with studies in the literature that identified malnutrition as a consistent risk factor for PI development [2,11,24].

The presence of comorbidity was independently associated with new PI development (OR = 2.327; 95% CI 1.016–5.330; *p* = 0.046), a finding that supports the concept of cumulative physiological vulnerability [25,26]. Although the Charlson Comorbidity Index did not differ significantly across PI groups in this cohort (*p* = 0.173), the presence of at least one comorbidity remained an independent predictor of new PI development in the multivariable model (OR = 2.327; 95% CI: 1.016–5.330; *p* = 0.046). This finding suggests that the binary distinct ion between comorbid and non-comorbid patients may capture clinically relevant physiological vulnerability more effectively than composite burden scores in this population.

Model discrimination was acceptable (AUC = 0.756). This AUC is comparable to the reported values for the Braden scale in ICU populations. Hyun et al. reported an AUC of 0.672 (95% CI 0.663–0.683) for the Braden scale [27], whereas a meta-analysis by Wei et al. reported a pooled AUC of 0.78 (95% CI: 0.716–0.846) [28]. The AUC obtained in our multivariate approach falls within this range, indicating clinically useful discrimination.

Moreover, Hosmer–Lemeshow testing (*p* = 0.285) indicated good model calibration. However, the model explained approximately 25% of the variance (Nagelkerke R^2^ ≈ 0.25), which underscores that PI development is a multifactorial, time-dependent process in which environmental and care-related variables likely also play important roles.

Although the Braden scale is a validated PI risk instrument and differed significantly between groups, it was not included in the primary multivariable model for several reasons. First, the Braden total score demonstrated a strong correlation with GCS (r = 0.828; *p* < 0.001), indicating a high degree of conceptual overlap, particularly through the sensory perception subscale. Including both variables simultaneously would risk multicollinearity and redundancy. Second, in a sensitivity analysis in which Braden score was added to Model A alongside GCS, NRS-2002, and comorbidity, Braden score was not independently associated with new PI development (OR = 0.877; 95% CI: 0.714–1.077; *p* = 0.212), and the effects of the other predictors and the overall model performance remained essentially unchanged (AUC = 0.760). These findings suggest that the predictive information carried by the Braden scale is largely captured by GCS and NRS-2002 within this model. The Braden scale nevertheless retains clinical value as a structured bedside risk-assessment tool and is recommended as a complement to the predictors identified in this study.

### 4.2. Clinical Improvement Model (Model B)

In patients with a PI at admission, the only variable independently associated with clinical improvement was the NRS-2002 score. In the multivariable exploratory model, NRS-2002 score (OR = 0.450; 95% CI: 0.274–0.739; *p* = 0.002) and age (OR = 1.058; 95% CI: 1.008–1.111; *p* = 0.022) were independently associated with clinical improvement. Albumin and Braden score did not contribute significantly to the model (both *p* > 0.05). The emergence of age as an independent predictor is consistent with evidence that older patients face greater barriers to PI healing due to frailty, impaired tissue perfusion, and diminished regenerative capacity [26]. This finding indicates that nutritional risk is independently associated with not only incident PI development but also the healing trajectory of existing lesions.

The explanatory power of the model was moderate (Nagelkerke R^2^ = 0.281), indicating that the healing process cannot be fully explained by a single clinical parameter. However, Hosmer–Lemeshow testing (*p* = 0.071) supported acceptable calibration of the model.

### 4.3. Clinical Implications

Each one-point increase in GCS was associated with a lower likelihood of PI development. NRS-2002 risk level was independently associated with PI development.

Among patients with an existing PI at admission, NRS-2002 was the only independent predictor of clinical improvement. This distinction indicates that baseline PI risk and PI prognosis are not interchangeable constructs.

## 5. Conclusions

In ICU patients, lower GCS, higher NRS-2002 risk level, and presence of comorbidity were independent predictors of new PI development in multivariable analysis. In patients who already had PIs at admission, NRS-2002 and age were independently associated with clinical improvement in an exploratory multivariable model, with NRS-2002 emerging as the dominant predictor. These findings suggest that nutritional risk influences not only PI incidence but also prognosis. The differing predictors for incident PIs versus healing imply that the pathophysiological dynamics underlying PI development and recovery may differ.

These results suggest that integrated assessment using GCS and NRS-2002 alongside APACHE II and Braden scale may enable more targeted risk stratification in ICU patients. Prospective validation studies are required to evaluate the model’s utility as a clinical decision-support tool.

This was a single-center study with a retrospective, observational design. Data were obtained from routine clinical records. Consequently, the exact timing of changes in PI stage could not always be ascertained. The retrospective design limited our ability to analyze the dynamic relationship between risk scores (GCS, Braden, NRS-2002) and clinical course over time. The exclusion of patients with an ICU stay of less than five days was intended to ensure sufficient observation time for PI development and staging, as PIs may require several days to become clinically apparent. However, this criterion may have introduced selection bias toward more severely ill patients, and PIs already present on admission in the shorter-stay group—particularly among those transferred from the emergency department—may not have been captured. A sensitivity analysis comparing patients with ICU stays of less than five days versus five or more days was performed; patients with shorter stays had significantly higher GCS scores and albumin levels (both *p* < 0.001), and a lower rate of new PI development (17.1% vs. 39.6%; *p* < 0.001), suggesting that they represented a clinically distinct and less severely ill subgroup. These findings support the appropriateness of the five-day threshold for identifying a more homogeneous, at-risk population, while acknowledging that patients discharged or deceased before five days are not represented in the present analysis.

Variables related to preventive nursing care processes—including frequency of patient repositioning, use of pressure-redistributing support surfaces, and adherence to standardized PI prevention bundles—were not systematically recorded in the hospital information system and could not be included in the analyses. The absence of these data limits the ability to distinguish the independent contribution of care-process variables from patient-level risk factors. Future prospective studies should incorporate standardized nursing care documentation to allow a more comprehensive evaluation of modifiable PI risk.

The retrospective design precluded the collection of standardized, time-stamped data on PI onset and healing milestones. Consequently, time-to-event analyses could not be performed, and PI development and clinical improvement were analyzed as binary outcomes. Future prospective studies incorporating systematic daily PI staging documentation would allow survival analysis methods to be applied, providing a more nuanced characterization of the temporal dynamics of PI development and recovery in the ICU.

## Figures and Tables

**Table 1:** Demographic, Clinical, and Laboratory Characteristics of Patients Included in the Study (n = 220).

Continuous Variables	Median (IQR)
Length of hospitalization (days)	19 (12–34)
Length of ICU stay (days)	10 (6–18.75)
Age, (years)	74 (64–85)
Charlson Comorbidity Index	7 (6–9)
Braden score	10 (6–13)
APACHE II	24 (20–27)
Glasgow Coma Scale	9 (4–12)
NRS-2002	4 (3–5)
BMI (kg/m^2^)	26.27 (23.87–29.38)
Albumin g/L	30.7 (27.35–34.8)
D-Dimer (mg/L)	1.58 (0.76–3.55)
Lactate (mmol/L)	1.7 (1.3–2.6)
CRP (mg/L)	73 (36–123)
Categorical Variables	n (%)
Sex	
Female	98 (44.5)
Male	122 (55.5)
Source unit on admission	
Emergency Department	118 (53.6)
Inpatient Ward	22 (10)
Level 1 ICU	22 (10)
Level 2 ICU	44 (20)
Level 3 ICU	12 (5.5)
Palliative unit	2 (0.9)
Comorbidities *	
Malignancy	30 (13.6)
Cardiovascular/Metabolic	112 (50.9)
Respiratory	51 (23.2)
No Comorbidity	59 (26.8)
Pressure Injury group	n (%)
No PI/None developed	84 (38.2)
New PI developed	55 (25)
Existing PI worsened/unchanged	61 (27.7)
Existing PI improved/regressed	20 (9.1)

Note. Continuous variables are presented as median (IQR) and categorical variables as n (%). * Comorbidities are multiple-response; therefore, percentages may exceed 100%. IQR, interquartile range; APACHE II, Acute Physiology and Chronic Health Evaluation II; NRS-2002, Nutritional Risk Screening 2002; BMI, Body Mass Index; CRP, C-reactive protein; ICU, Intensive Care Unit.

**Table 2:** Comparison of Continuous Variables According to PI Groups.

Variable	No PI/None Developed (n = 84)	New PI Developed (n = 55)	Existing PI Worsened/Unchanged (n = 61)	Existing PI Improved/Regressed (n = 20)	*p*
Age, (years)	70 (62.3–79.8)	73 (60–85)	79 (67.5–87)	84.5 (74.3–90.3)	**0.003**
Charlson Comorbidity Index	7 (6–8)	7 (6–9)	8 (5–10)	7 (6–8)	**0.173**
APACHE II	23 (18–27)	24 (20–27)	25 (21–29)	24 (20–25.8)	0.362
GCS	12 (8.3–14.8)	8 (4–12)	8 (4–12)	9 (4–12)	**<0.001**
NRS-2002	4 (3–4)	4 (3–5)	5 (4–6)	4 (3–4.8)	**<0.001**
Braden	12 (9–15)	9 (6–12)	9 (6–11.5)	9.5 (6–13)	**<0.001**
Albumin (g/L)	32.75 (29.1–35.7)	30.3 (27.1–36)	28.8 (26.3–32.9)	30.5 (27.2–31.7)	**0.002**
D-Dimer (mg/L)	1.07 (0.6–2.9)	1.71 (0.9–3)	2.52 (1.1–5.2)	1.15 (0.7–2.6)	**0.006**
CRP (mg/L)	81.3 (36.1–134.2)	85 (56–145)	66 (33.9–102)	51.5 (35–101)	0.236
Lactate (mmol/L)	1.7 (1.2–2.7)	1.7 (1.5–2.4)	1.9 (1.4–2.7)	1.7 (1.2–2.8)	0.716
Length of hospitalization (days)	14 (10–25.8)	20 (13–40)	26 (14.5–42)	18.5 (11.3–36.3)	**0.002**
Length of ICU stay (days)	9 (6–16)	11 (7–21)	12 (7–23.5)	7.5 (6–17.5)	0.196

Note. Data are presented as median (IQR). Kruskal–Wallis test was used for intergroup comparisons. Dunn–Bonferroni post hoc analysis was applied to variables that reached statistical significance. A two-tailed *p* < 0.05 was considered statistically significant. Abbreviations: PI, pressure injury; APACHE II, Acute Physiology and Chronic Health Evaluation II; GCS, Glasgow Coma Scale; NRS-2002, Nutritional Risk Screening 2002; CRP, C-reactive protein; IQR, interquartile range. Bold values indicate statistical significance (*p* < 0.05).

**Table 3:** Dunn–Bonferroni Post hoc Comparisons According to PI Groups.

Variable	Group Comparison	Adj. *p*
Age	1–4	0.008
GCS	2–1	<0.001
	3–1	0.002
NRS-2002	1–3	<0.001
	4–3	0.003
	2–3	<0.001
Braden	3–1	<0.001
	2–1	<0.001
Albumin	3–1	0.001
D-Dimer	1–3	0.005
Length of hospitalization	1–3	0.001

Note. 1, No PI/None developed; 2, New PI developed; 3, Existing PI worsened/unchanged; 4, Existing PI improved/regressed. Adjusted *p*-values are Bonferroni corrected. A two-tailed *p* < 0.05 was considered statistically significant. Abbreviations: PI, pressure injury; GCS, Glasgow Coma Scale; NRS-2002, Nutritional Risk Screening 2002; Adj. *p*, adjusted *p* value.

**Table 4:** Comparison of Categorical Variables According to PI Groups.

Variable	No PI/None Developed (n = 84)	New PI Developed (n = 55)	Existing PI Worsened/Unchanged (n = 61)	Existing PI Improved/Regressed (n = 20)	*p*
Comorbidities					0.050
No	21 (25)	21 (38.2)	10 (16.4)	7 (35)	
≥1	63 (75)	34 (61.8)	51 (83.6)	13 (65)	
Source unit on admission					0.025 *
Emergency Department	49 (58.3)	38 (69.1)	23 (37.7)	8 (40)	
Inpatient Ward	12 (14.3)	1 (1.8)	7 (11.5)	2 (10)	
Level 1 ICU	8 (9.5)	5 (9.1)	7 (11.5)	2 (10)	
Level 2 ICU	10 (11.9)	9 (16.4)	18 (29.5)	7 (35)	
Level 3 ICU	5 (6)	2 (3.6)	5 (8.2)	0 (0)	
Palliative unit	0 (0)	0 (0)	1 (1.6)	1 (5)	
Need for sedation					**0.014**
Yes	45 (53.6)	43 (78.2)	33 (54.1)	10 (50)	
No	39 (46.4)	12 (21.8)	28 (45.9)	10 (50)	
Need for vasopressors					**0.037**
Yes	47 (56)	43 (78.2)	42 (68.9)	11 (55)	
No	37 (44)	12 (21.8)	19 (31.1)	9 (45)	
Braden Risk Level					0.015 *
High risk	47 (56)	42 (76.4)	49 (80.3)	14 (70)	
Moderate risk	33 (39.3)	13 (23.6)	12 (19.7)	6 (30)	
Mild risk	4 (4.8)	0 (0)	0 (0)	0 (0)	
NRS-2002 Risk Level					**<0.001**
Low risk	31 (36.9)	24 (43.6)	7 (11.5)	9 (45)	
At risk	40 (47.6)	13 (23.6)	16 (26.2)	6 (30)	
High risk	13 (15.5)	18 (32.7)	38 (62.3)	5 (25)	

Note. Data are presented as n (%). When expected cell frequencies were low, analyses used Monte Carlo simulation. *p*-values derived by Monte Carlo are marked with (*). A two-tailed *p* < 0.05 was considered statistically significant. Abbreviations: ICU, Intensive Care Unit; Level 1 ICUs provide basic monitoring and support; Level 2 ICUs are equipped for mechanical ventilation and advanced hemodynamic monitoring; Level 3 ICUs provide the full spectrum of organ support including renal replacement therapy and advanced mechanical circulatory assistance; NRS-2002, Nutritional Risk Screening 2002. Bold values indicate statistical significance (*p* < 0.05).

**Table 5:** Factors Independently Associated with the Development of New PIs in Patients Without PIs on Admission (Model A, n = 139).

Variable	OR (95% CI)	*p*
GCS	0.824 (0.746–0.911)	<0.001
Comorbidity (Yes)	2.327 (1.016–5.330)	0.046
NRS-2002 Risk Level		0.028 (overall *p*)
Low risk vs. High risk	0.263 (0.095–0.728)	0.010
At risk vs. High risk	0.608 (0.230–1.607)	0.316

Note. Dependent variable: New PI development (0 = No, 1 = Yes). Reference categories: High risk for NRS-2002, none for comorbidity. Model calibration was evaluated using the Hosmer–Lemeshow test (*p* = 0.285). The model explained approximately 25% of the variance in new PI development (Nagelkerke R^2^ = 0.254). ROC analysis indicated acceptable discrimination (AUC = 0.756). There was no multicollinearity among the independent variables (all VIF values ≈ 1). n = 139; number of events = 55. Abbreviations: GCS, Glasgow Coma Scale; OR, odds ratio; CI, confidence interval; NRS-2002, Nutritional Risk Screening 2002; ROC, receiver operating characteristic; AUC, area under the curve; VIF, variance inflation factor.

**Table 6:** Factors Independently Associated with Clinical Improvement in Patients with PIs on Admission (Model B—Exploratory Model, n = 81).

Variable	OR (95% CI)	*p*
NRS-2002 score	0.450 (0.274–0.739)	0.002
Albumin	1.025 (0.907–1.159)	0.690
Braden score	1.013 (0.848–1.211)	0.883
Age	1.058 (1.008–1.111)	0.022

Dependent variable: Clinical improvement (0 = No, 1 = Yes). Model B is an exploratory model. Model calibration was acceptable (Hosmer–Lemeshow *p* > 0.05). The model explained approximately 28% of the variance in clinical improvement (Nagelkerke R^2^ = 0.281). OR, odds ratio; CI, confidence interval.

## Data Availability

The data are not publicly available due to patient privacy and institutional restrictions but may be available from the corresponding author upon reasonable request.

## Abbreviations

The following abbreviations are used in this manuscript:

APACHE II	Acute Physiology and Chronic Health Evaluation II
BMI	Body Mass Index
PI	Pressure Injury
CRP	C-Reactive Protein
CI	Confidence Interval
GCS	Glasgow Coma Scale
ICU	Intensive Care Unit
NRS-2002	Nutritional Risk Screening 2002
OR	Odds Ratio
IQR	interquartile range
kg/m^2^	kilograms per square meter
g/L	grams per liter
mg/L	milligrams per liter
mmol/L	millimoles per liter
